# Melatonin, programmed death ligand-1, programmed death ligand-1, and cancer: imagine beyond the future?

**DOI:** 10.1590/1806-9282.0713EDIT

**Published:** 2025-05-02

**Authors:** José Maria Soares, Ilker Sengul, Demet Sengul

**Affiliations:** 1 Universidade de São Paulo, Hospital das Clínicas, Faculdade de Medicina, Departamento de Obstetrícia e Ginecologia, Disciplina de Ginecologia, Laboratório de Ginecologia Estrutural e Molecular (LIM-58) – São Paulo (SP), Brazil.; 2 Giresun University, Faculty of Medicine, Division of Endocrine Surgery – Giresun, Turkey.; 3 Giresun University, Faculty of Medicine, Department of General Surgery – Giresun, Turkey.; 4 Giresun University, Faculty of Medicine, Department of Pathology – Giresun, Turkey.

Melatonin (N-acetyl-5-methoxytryptamine) (MT) is an indolamine and a neurohormone that is primarily synthesized and secreted mainly by the pinecone-shaped gland of the cerebrum, called the conarium or epiphysis cerebri, from amino acid tryptophan. MT, which has various biologic effects, like regulation of circadian rhythm, as well as antioxidant, anti-aging, and antitumor activities, was first isolated in 1958 from the bovine pineal gland by Maliki et al.^1^ Furthermore, the 17th-century philosopher René Descartes hypothesized the pineal gland of the brain, representing the location of the *Homo sapiens* soul, which paleontologists described it as an ancestral "third eye." The third eye, per se, remains poorly understood, and modern psychology declares perception beyond physical visual function^2,3^.

This "eye-associated" chemical messenger, per se, ensures high precision in recognizing the night period. It is an endocrine marker for darkness and regulates circadian rhythm and the sleep–wake cycle. Moreover, it can be reproduced by other vital organs, such as the brain, thyroid, lungs, gastrointestinal tract, liver, and reproductive and immune systems. Notably, it is present in mucus, saliva, breast milk, amniotic fluid, Graff follicle, sperm, urine, etc^1,4^. MT has been reported to possess significant antioxidant, anti-inflammatory, antiproliferative, pro-apoptotic, anti-angiogenic, and antimetastatic immunomodulatory properties^5,6^. Based on the immunoregulatory properties of MT, it has been researched as a therapeutic option for many autoimmune diseases, which attains its impact through the MT receptors type 1 and type 2 (MT1 [Mel_1a_] and MT2 [Mel_1b_]) of the membrane-bound receptors. Additionally, the presence of the MT1 receptor in the papillon gland has been proven to resemble the contingency of MT's impact on the thyroid activity and reproduction of hormonal processes. However, the MT3 receptor, the third membrane-bound MT binding site, was theorized as a biological and was detected to, in fact, be the cytosolic enzyme, quinone reductase II (NQO2)^7^. A single-nucleotide polymorphism of MTNR1A, coding the MT1 protein, gave liaison to a susceptibility of Graves’ disease and thyroid autoantibody formation, which led to the contingency of the development of autoimmune thyroid disease in thyroidology^8-10^.

Programmed cell death-1 (PCD-1) is a crucial immunological checkpoint receptor. It is preferably expressed in activated cells involving T, B, dendritic (DC), natural killer (NK), and T reg. In addition to this, PCD-1 is linked to augmented Treg-cell proliferation and enhanced immunosuppressive function. As such, the epithelial, endothelial, hematopoietic, and tumor cells can produce programmed death ligand-1 (PD-L1) and programmed death ligand-2 (PD-L2) ligands. They are regulated by a couple of inflammatory cytokines involving interferon-gamma (IFN-γ), released by activated T and NK cells. Of note, oncogenes might be vital in promoting PD-L1 expression, unlike PD-L2^11-13^. PD-L1 messenger RNA (mRNA) transcription augments with the activation of the MEK/ERK^14-17^ kinase through nuclear factor-kappa B, essential for its toll-like receptor (TLR)-mediated regulation. Furthermore, IFN receptors 1 and 2 are connected to regulating the PD-L1 expression of interferon regulatory factor-(IRF-1), which is another way through the Janus kinase (Jak)/signal transducer(s) and activator(s) of the transcription (STAT) pathway. The phosphatidylinositol 3 kinase (PI3K)/serine/threonine protein kinase B (PKB, also known as AKT) pathway^14-17^ plays a permissive role in PD-L1 transcription through the phosphorylation of rapamycin's mammalian target. Notably, it might also up-regulate PD-L1 expression in response to the IFN-mediated activation of Jak/STAT^18^. To date, PD-L1 is weakly expressed in normal tissues, though it is overexpressed in many tumor cells. It was indicated that this leads to PD-L1 being an immunotherapeutic target^19^. The PD-L1-positive tumors were reported to respond to the treatment noticeably better than those PD-L1 negative. PD-L1 with a high expression in tumor cells was emphasized with a better response to nivolumab in recurrent head-and-neck cancer or renal cell carcinoma, like pembrolizumab in advanced non-small cell lung cancer (NSCLC). The interplay of PD-L1 on the membrane of cancer cells with PCD-1 expression on T cells provokes T cell exhaustion and attenuation of the succeeding immune reaction, skipping immune surveillance. Up to now, the mechanism of PD-L1 and -L2 regulation in tumorigenesis immune escape remains largely inscrutable^20-22^. The PD1 receptor might be located on both CD8+ and CD4+ T cells. At the same time, PD-L1 is expressed by activated T cells, tumor-infiltrating macrophages or fibroblasts, and ovarian cancer cells, which may impose upon the immune response against the tumorigenesis^22,23^. A growing interest in the possibility of employing immunotherapy in cases with gynecological cancers has led to the advancement of a large number of clinical trials testing immunotherapy as monotherapy and in combination with other strategies, such as chemotherapy, targeted agents, or both. As such, cancer immunotherapy targeting PCD-1 or PD-L1 has proven effective in causing durable antitumor immune responses with less toxicity in many tumors, including gynecological cancer^22-26^.

MT was reported in order to enhance the antitumor activity of macrophages, suppressed by exosomes from gastric cancer cells, which was achieved through the regulation of PD-L1 in macrophages via the modulation of the associated microRNAs in the cancer-derived exosomes^27^. Moreover, MT treatment announced significant attenuation of cell viability in companies that trigger cell apoptosis in KRAS-mutant NSCLC cell lines, embracing A549, H460, and LLC1 cells. The lung cancer cells possessing the KRAS mutation exhibited an excelsior level of PD-L1. Nevertheless, MT therapy downregulated PD-L1 expressions on a large scale in both the presence and absence of IFN-γ stimulation^28^.

Finally, though MT has demonstrated a broad spectrum of anticancer impacts and PCD-1 might be a key immune checkpoint receptor that mainly acts on activated T, B, DC, NK, and T reg cells, and checkpoint blockades are registered for the treatment of various cancers, accurately modulating tumor immunity remains largely unknown today. This issue merits further investigation—*post tenebras lux*.

## References

[1] Maliki OF, Alagbon AI, Ibitoye CM, Olayaki LA (2021). Melatonin and vitamin C modulate cassava diet-induced alteration in reproductive and thyroid functions. Niger J Exp Clin Biosci.

[2] Lane R (2023). Of sight, and insight. Lancet.

[3] Soares JM, Kesicioglu T, Sengul D, Sengul I (2023). Of sight, and insight into melatonin's role in breast cancer?. Rev Assoc Med Bras (1992).

[4] Stanojlović O, Šutulović N (2018). Cirkadijalni sistem - mreža neurona sa suprahijazmatičnim jedrom na vrhu hijerarhijske organizacije. Med Podml.

[5] Wang SW, Tai HC, Tang CH, Lin LW, Lin TH, Chang AC (2021). Melatonin impedes prostate cancer metastasis by suppressing MMP-13 expression. J Cell Physiol.

[6] Farhood B, Goradel NH, Mortezaee K, Khanlarkhani N, Najafi M, Sahebkar A (2019). Melatonin and cancer: from the promotion of genomic stability to use in cancer treatment. J Cell Physiol.

[7] Slominski RM, Reiter RJ, Schlabritz-Loutsevitch N, Ostrom RS, Slominski AT (2012). Melatonin membrane receptors in peripheral tissues: distribution and functions. Mol Cell Endocrinol.

[8] Lin JD, Fang WF, Tang KT, Cheng CW (2019). Effects of exogenous melatonin on clinical and pathological features of a human thyroglobulin-induced experimental autoimmune thyroiditis mouse model. Sci Rep.

[9] Sengul I, Sengul D, Pelikán A (2020). Iodine-induced hyperthyroidism: Do you mind?. Sanamed.

[10] Sengul D, Sengul I, Soares JM (2022). Repercussion of thyroid dysfunctions in thyroidology on the reproductive system: conditio sine qua non?. Rev Assoc Med Bras (1992).

[11] Bardhan K, Anagnostou T, Boussiotis VA (2016). The PD1:PD-L1/2 pathway from discovery to clinical implementation. Front Immunol.

[12] Yearley JH, Gibson C, Yu N, Moon C, Murphy E, Juco J (2017). PD-L2 expression in human tumors: relevance to anti-PD-1 therapy in cancer. Clin Cancer Res.

[13] Shin SJ, Jeon YK, Kim PJ, Cho YM, Koh J, Chung DH (2016). Clinicopathologic analysis of PD-L1 and PD-L2 expression in renal cell carcinoma: association with oncogenic proteins status. Ann Surg Oncol.

[14] Sengul I, Sengul D (2010). Ischemic preconditioning and postconditioning in cardiovascular surgery. Cumhuriyet Med J.

[15] Sengul D, Sengul I (2021). Connection of reactive oxygen species as an essential actor for the mechanism of phenomena; ischemic preconditioning and postconditioning: come to age or ripening?. North Clin Istanb.

[16] Sengul I, Sengul D (2010). Potasyum–ATP kanalları ve reperfüzyon hasarı azaltıcı koşullanmalar. Nobel Med.

[17] Sengul I, Sengul D, Guler O, Hasanoglu A, Urhan MK, Taner AS (2013). Postconditioning attenuates acute intestinal ischemia-reperfusion injury. Kaohsiung J Med Sci.

[18] Boussiotis VA, Chatterjee P, Li L (2014). Biochemical signaling of PD-1 on T cells and its functional implications. Cancer J.

[19] Tsushima F, Iwai H, Otsuki N, Abe M, Hirose S, Yamazaki T (2003). Preferential contribution of B7-H1 to programmed death-1-mediated regulation of hapten-specific allergic inflammatory responses. Eur J Immunol.

[20] Świderska J, Kozłowski M, Kwiatkowski S, Cymbaluk-Płoska A (2021). Immunotherapy of ovarian cancer with particular emphasis on the PD-1/PDL-1 as target points. Cancers (Basel).

[21] Otoshi T, Nagano T, Tachihara M, Nishimura Y (2019). Possible biomarkers for cancer immunotherapy. Cancers (Basel).

[22] Gutic B, Bozanovic T, Mandic A, Dugalic S, Todorovic J, Stanisavljevic D (2023). Programmed cell death-1 and its ligands: current knowledge and possibilities in immunotherapy. Clinics (Sao Paulo).

[23] Leary A, Tan D, Ledermann J (2021). Immune checkpoint inhibitors in ovarian cancer: where do we stand?. Ther Adv Med Oncol.

[24] Bray F, Ferlay J, Soerjomataram I, Siegel RL, Torre LA, Jemal A (2018). Global cancer statistics 2018: GLOBOCAN estimates of incidence and mortality worldwide for 36 cancers in 185 countries. CA Cancer J Clin.

[25] Eymerit-Morin C, Ilenko A, Gaillard T, Varinot J, Compérat E, Bendifallah S (2021). PD-L1 expression with QR1 and E1L3N antibodies according to histological ovarian cancer subtype: a series of 232 cases. Eur J Histochem.

[26] Michielin O, Akkooi ACJ, Ascierto PA, Dummer R, Keilholz U, ESMO Guidelines Committee (2019). Cutaneous melanoma: ESMO clinical practice guidelines for diagnosis, treatment and follow-up†. Ann Oncol.

[27] Wang K, Cai R, Fei S, Chen X, Feng S, Zhang L (2023). Melatonin enhances anti-tumor immunity by targeting macrophages PD-L1 via exosomes derived from gastric cancer cells. Mol Cell Endocrinol.

[28] Chao YC, Lee KY, Wu SM, Kuo DY, Shueng PW, Lin CW (2021). Melatonin downregulates PD-L1 expression and modulates tumor immunity in KRAS-mutant non-small cell lung cancer. Int J Mol Sci.

